# Assessment of Clinical Decision Support System Efficiency in Spinal Neurosurgery for Personalized Minimally Invasive Technologies Used on Lumbar Spine

**DOI:** 10.17691/stm2021.13.5.02

**Published:** 2021-10-29

**Authors:** V.А. Byvaltsev, А.А. Kalinin

**Affiliations:** Professor, Head of the Department of Neurosurgery and Innovative Medicine Irkutsk State Medical University, 1 Krasnogo Vosstaniya St., Irkutsk, 664003, Russia;; Chief of Neurosurgery Center Road Clinical Hospital, 10 Botkin St., Irkutsk, 664005, Russia;; Professor, Department of Traumatology, Orthopedics and Neurosurgery Irkutsk State Medical Academy for Postgraduate Education, 100 Yubileyny Microdistrict, Irkutsk, 664049, Russia; Associate Professor, Department of Neurosurgery and Innovative Medicine Irkutsk State Medical University, 1 Krasnogo Vosstaniya St., Irkutsk, 664003, Russia;; Neurosurgeon, Neurosurgery Center Road Clinical Hospital, 10 Botkin St., Irkutsk, 664005, Russia

**Keywords:** degenerative lumbar diseases, minimally invasive spinal neurosurgery, machine learning, artificial intelligence, clinical decision support systems

## Abstract

**The aim of the study** was to assess clinical decision support system (CDSS) in spinal surgery for personalized minimally invasive technologies on lumbar spine.

**Materials and Methods:**

The prospective study involved 59 patients operated on using CDSS based on a personalized surgical algorithm considering patient-specific parameters of lumbar segments. Among them, 11 patients underwent total disk replacement (TDR), 25 and 23 patients had minimally invasive (MI-TLIF) and open (O-TLIF) dorsal rigid stabilization, respectively, according to an original technology. The comparative analysis was carried out using retrospective findings of 196 patients operated on involving TDR (n=42), MI-TLIF (n=79), and O-TLIF (n=75). The efficiency of CDSS medical algorithms was assessed by pain syndrome in the lumbar spine and lower limbs, as well as by patients’ functional status on discharge according to ODI, 3 and 6 months after the operation.

**Results:**

The comparison by gender characteristics and anthropometric data revealed no significant intergroup differences among the groups under study (p>0.05). Intergroup analysis of functional status by ODI, pain intensity in lower limbs and lumbar spine showed better clinical outcomes in patients operated using CDSS compared to a retrospective group (p<0.05): 6 months after TDR and O-TLIF, and 3 months after MI-TLIF.

**Conclusion:**

The study findings demonstrated high efficiency of CDSS developed for personalized surgical treatment of patients with degenerative lumbar spine diseases taking into consideration individual biometric parameters of lumbar segments.

## Introduction

Degenerative changes in anatomical elements of lumbar spine are frequently accompanied by developing compression and/or pseudoradicular clinical presentations [1]. A morphostructural cascade of the changes is characterized by gradual degeneration of intervertebral discs (IVD) and facet joints (FJ) [2]. A variety of neurological manifestations, functional impairments of spinal units, as well as the intensity of pathological alterations in the anterior and posterior supporting complexes in degenerative lumbar diseases promote the development of various approaches in spinal surgery: from percutaneous puncture to extended decompressive stabilizing techniques [3].

Success in spinal surgeries directly depends on the elimination of clinical symptoms and recovery of normal biomechanics of the operated segments [4]. In addition, the lack of unique medical and diagnostic algorithms in vertebrology, high variability of surgical decisions, a wide range of morphological substrates specifying neurological disorders determine surgeon’s subjective decision making, and subsequently, rather high percentage of poor postoperative outcomes [5, 6]. Moreover, the above-mentioned circumstances involve heavy cost loading on healthcare system in the form of readmissions, reoperations, as well as perioperative complications, with long-term or permanent total disability of operated patients [7].

A methodology aimed at improvement of surgical outcomes in patients with degenerative lumbar diseases is based on determining objective clinical and anatomical structural parameters necessary to develop personalized surgical approaches, as well as on developing the criteria for adverse perioperative sequelae prediction [8, 9].

One of the techniques enabling to reduce an error rate in management and diagnostics in various medical fields is clinical decision support system (CDSS) based on processing large amounts of information and research findings [10]. This innovative approach is related to the use of machine learning (ML) and artificial intelligence (AI) [11]. Such technologies are currently successfully used in healthcare to support clinical decision making and patients’ screening after surgical interventions and nonsurgical treatment [12]. Thus, currently, a promising direction in medicine including spinal neurosurgery is the development of computer-aided systems using AI and ML, which enable to predict treatment results based on medical and diagnostic algorithms and mathematical calculations [13]. Potential application of the systems consists in better visualization of pathology, diagnostic accuracy improvement, and replacement of doctors’ routine work [14]. Despite an increasing number of publications about AI and ML in different fields of current medicine, CDSS development in spinal surgery is in the initial stage.

Neurosurgery Center of Road Clinical Hospital (Irkutsk, Russia) since 2020 has been using software developed on the basis of a large number of research findings (databases involving over 12,000 patients operated over the last 15 years) on using various surgical approaches to treat patients with degenerative diseases of lumbar spine depending on individual morphostructural characteristics of functional spinal units. Surface data on using CDSS in spinal surgery were an impulsive moment to implement the research project.

**The aim of the study** was to assess clinical decision support system in spinal surgery for personalized minimally invasive technologies on lumbar spine.

## Materials and Methods

### Background of clinical decision support system development in spinal neurosurgery

**Figure 1.**
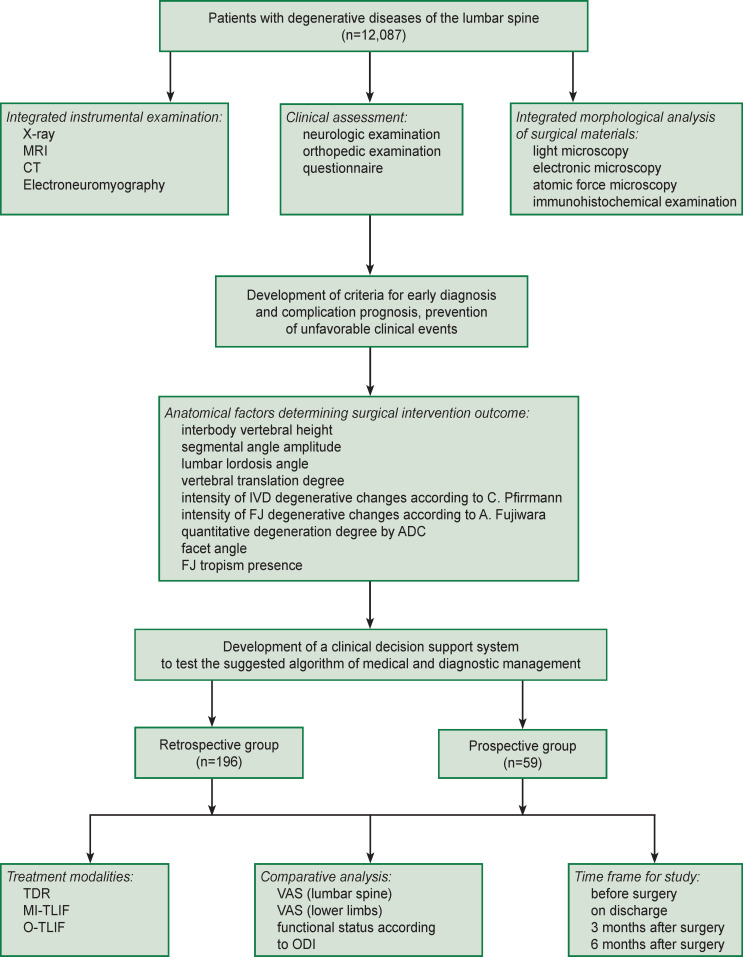
Study design flowchart Here: IVD — intervertebral disc; FJ — facet joint; ADC — apparent diffusion coefficient

Within the framework of the previous researches (grant of President of the Russian Federation “Study of connective tissue nanostructural organization change model under laser radiation” (MD-6662.2012.7), Russian Science Foundation grant “Molecular signal cascades and their effect on nutritive transport via intercellular matrix for intervertebral disc regeneration” (project No.15-15-30037), state contract No.620-NIOKTR/1800/2625-EA/19 “Surgical treatment optimization of degenerative lumbar spine diseases in Irkutsk region” dated June 26, 2019), as well as the use of the array data of patients with degenerative diseases of the lumbar spine (n=12,087), who underwent treatment in Neurosurgery Center, Road Clinical Hospital (Irkutsk, Russia) in 2005–2020, we retrospectively analyzed the findings of an integrated clinical laboratory survey of the patients (Figure 1). From the cohort, by a random sampling technique, we chose those operated with the help of total disk replacement (ТDR, n=42), minimally invasive transforaminal interbody fusion (MI-TLIF, n=79), and open transforaminal interbody fusion (O-TLIF, n=75). Clinical and laboratory data (Table 1) were studied for prognosis and prevention of poor clinical outcomes.

**Table 1:** Clinical data of retrospective groups, Me [Q1; Q3]

Parameter	ТDR (n=42)		p	MI-TLIF (n=79)		p	O-TLIF (n=75)		p
	Good outcomes	Poor outcomes		Good outcomes	Poor outcomes		Good outcomes	Poor outcomes	
	(n=35)	(n=7)		(n=64)	(n=15)		(n=62)	(n=13)	
ODI after 24 months (points)	6 [6; 8]	16 [16; 18]	0.008	8 [6; 8]	26 [20; 28]	0.001	8 [8; 10]	32 [28; 36	0.003
VAS after 24 months (mm):									
lumbar spine	6 [5; 8]	14 [14; 16]	0.007	9 [8; 10]	24 [22; 26]	0.009	10 [10; 12]	29 [27; 30]	0.001
lower limbs	4 [3; 5]	16 [14; 18]	0.006	6 [5; 7]	22 [21; 23]	0.002	7 [7; 8]	24 [22; 25]	0.001
Linear translation (mm):									
before surgery	2 [1; 4]	6 [5; 7]	0.001	7 [4; 11]	3 [2; 3]	0.001	7 [5; 10]	3 [2; 4]	0.003
after surgery	1 [1; 2]	2 [1; 2]	0.005	2 [1; 3]	5 [4; 7]	0.006	2 [1; 3]	6 [4; 8]	0.001
Sagittal angulation (degrees):									
before surgery	4 [3; 5]	7 [6; 8]	0.002	8 [6; 9]	3 [2; 3]	0.005	8 [6; 9]	3 [2; 4]	0.004
after surgery	4 [4; 5]	3 [3; 5]	0.001	2 [1; 2]	3 [3; 4]	0.009	1 [1; 2]	3 [3; 5]	0.008
General lordosis (degrees):									
before surgery	30 [28; 36]	30 [26; 38]	0.003	32 [28; 36]	32 [23; 38]	0.45	34 [29; 41]	30 [26; 39]	0.26
after surgery	54 [48; 64]	34 [32; 36]	0.007	52 [44; 66]	37 [32; 38]	0.006	56 [42; 68]	35 [31; 39]	0.006
Intervertebral disc height (mm):									
before surgery	10 [9; 13]	6 [5; 9]	0.001	6 [5; 8]	10 [9; 12]	0.007	4 [3; 7]	10 [8; 14]	0.003
after surgery	12 [10; 12]	10 [10; 12]	0.004	13 [11; 15]	11 [10; 13]	0.005	12 [11; 14]	10 [6; 11]	0.009
Apparent diffusion coefficient	1540	1050		1180	1320		670	1170	
before surgery (s/mm^2^)	[1280; 1760]	[800; 1150]	0.003	[980; 1230]	[1240; 1520]	0.001	[450; 930]	[1080; 1660]	0.002
Facet joint angulation before surgery (degrees)	50 [44; 59]	69 [62; 74]	0.003	70 [62; 78]	52 [48; 56]	0.006	69 [61; 82]	52 [49; 56]	0.008
Facet joint tropism before surgery	+/–	+/–	0.15	+	+/–	0.001	–	+	0.002
Degradation degree according to:									
C. Pfirmann	II (I; II)	III (III; IV)	0.22	III (III; IV)	III (III; V)	0.008	III (III; V)	III (II; III)	0.004
A. Fujiwara	I (I; II)	II (I; II)	0.22	III (II; III)	III (III; IV)	0.008	III (III; IV)	III (II; III)	0.004

The analysis of the effect of the instrumental parameters under study have on long-term clinical outcomes and the study of possible management optimization of patients with degenerative lumbar spine diseases showed that in a group of patients operated according to dynamic fixation technique (ТDR) good long-term outcomes by visual analogue scale (VAS) and Oswestry questionnaire (Oswestry Disability Index, ODI) were achieved at preoperative parameters of linear translation of vertebrae to a maximum of 4 mm, sagittal range of motion — less 6°, at interbody vertebral height decrease no more than 2/3 from the superjacent one, I–II degeneration degree of IVD according to C. Pfirrmann, apparent diffusion coefficient (ADC) being not less than 1240 s/mm^2^, I–II degeneration degree of IVD according to A. Fujiwara, facet angle of less 60°, regardless tropism. In subgroups of patients operated according to rigid stabilization (MI-TLIF and O-TLIF), minimal long-term outcomes by VAS and ODI were achieved at preoperative parameters of over 4-mm-linear vertebral translation, sagittal range of motion — not less than 6°, at interbody vertebral height decrease no more than 2/3 from the superjacent one, facet angle being over 60°. However, in case of III–IV degradation degree of IVD according to C. Pfirrmann, ADC was less than 1150 s/mm^2^, II–III degeneration degree of FJ according to A. Fujiwara, as well as no tropism, it is possible to perform minimally invasive rigid stabilization; in case of IV–V degeneration degree of IVD according to C. Pfirrmann, if ADC is less than 950 s/mm^2^, IV degeneration degree of FJ according to A. Fujiwara and the presence of tropism, it is reasonable to perform open vertebral fusion and transpedicular stabilization.

The recommendations on using a medical algorithm were developed to perform minimally invasive surgeries in patients with degenerative lumbar spine diseases based on an integral preoperative clinical and laboratory assessment [15].

### Description and application of clinical decision support system

CDSS was developed as a system for determining personalized surgical management of patients with degenerative diseases of lumbar spine based on leading instrumental features of anatomical characteristics of lumbar segments. As input data, we used primary data on patients, clinical and instrumental findings. Output reactions were treatment algorithms.

Computer-assisted system includes an electronic checklist, which has preoperative instrumental data on lumbar segments of patients with degenerative diseases. The software was developed using UMKB (United Medical Knowledge Base) — a semantic network structured on the basis of medical ontology and fuzzy logic principles, under the terms of the partner cooperation agreement with JSC “Sotsmedika”.

Using CDSS according to the suggested algorithm of a personalized surgical treatment considering individual parameters of lumbar segments, since September 2020, 59 patients have been operated on, they underwent ТDR (n=11), MI-TLIF (n=25), and O-TLIF (n=23). For rigid stabilization, we used original surgical techniques — MI-TLIF [16] and O-TLIF [17]. All patients involved in the prospective study had minimal follow-up — 6 months after surgery.

Inclusion criteria:

ineffective conservative treatment, long-term or recurrent pain syndrome, permanent neurologic deficiency from radicular pain to radiculopathy with peripheral pareses;

combination of radicular and pseudoradicular clinical presentation;

interbody vertebral height decrease for over 1/3 of the superjacent one;

according to neuroimaging — a single-level symptomatic degenerative lumbar spine disease.

Exclusion criteria:

central spinal stenosis;

spondylolisthesis with or without spondylolysis;

severe comorbidity;

osteoporosis (2.8 or more bone mineral density decrease according to Т-criterion (WHO, 1995));

necessity for significant sagittal balance correction;

necessity for surgery on two or more segments of the lumbar spine.

A comparative analysis of treating patients using CDSS and a control retrospective group was carried out on discharge, 3 and 6 months after surgery by pain syndrome level in the lumbar spine and lower limbs, as well as by functional status according to ODI.

The study was carried out in accordance with Declaration of Helsinki (2013) and approved by the Ethics Committee of Irkutsk State Medical University (Russia). An informed consent was obtained from each patient.

### Statistical data analysis

The study findings were statistically processed using Statistica 8.0 software. The distribution pattern of characteristics was assessed by normality tests: Shapiro–Wilk statistics, Kolmogorov– Smirnov test, Lilliefors test. Taking into account the significant differences (p<0.05), the distribution was considered different from normal, and to assess significant differences of the sampling populations we used nonparametric statistic criteria. Differences were considered significant if p<0.05. The findings were represented as median, values of the 1^st^ and 3^rd^ quartiles — Me [Q1; Q3]. For a comparative analysis, we used Mann–Whitney U-test and Wilcoxon criterion for nonparametric data, χ^2^ criterion — for binomial signs.

## Results

Table 2 represents the patient groups under study. No significant intergroup differences were revealed when comparing the groups by gender characteristics and anthropometric data (p>0.05). The highest frequency of surgical interventions was recorded in lower lumbar segments L_4_–L_5_ and L_5_–S_1_.

**Table 2:** Common data on patients’ groups under study

Parameter	ТDR		р	MI-TLIF		р	O-TLIF		р
	Prospective	Retrospective		Prospective	Retrospective		Prospective	Retrospective	
	(n=11)	(n=42)		(n=25)	(n=79)		(n=23)	(n=75)	
Age (years),									
Me [Q1; Q3]	34 [30; 39]	35 [32; 42]	0.54	36 [34; 41]	37 [33; 48]	0.31	45 [37; 55]	43 [35; 54]	0.68
Sex, n (%):									
male	8 (72.7)	29 (69)	0.81	16 (64)	57 (72.2)	0.43	14 (60.9)	51 (68)	0.52
female	3 (27.3)	13 (31)		9 (36)	22 (27.8)		9 (39.1)	24 (32)	
Body mass index,	24.5	25.6		26.4	26.1		27.0	26.5	
Me [Q1; Q3]	[23.0; 28.8]	[23.1; 29.6]	0.29	[23.7; 29.2]	[23.3; 29.6]	0.15	[24.1; 29.2]	[23.6; 29.9]	0.77
Localization of operated segments, n (%):									
L_2_–L_3_	—	—	—	—	—	—	—	1 (1.3)	—
L_3_–L_4_	—	1 (2.4)	—	2 (8)	9 (11.4)	—	2 (8.7)	8 (10.7)	—
L_4_–L_5_	4 (36.4)	17 (40.5)	—	9 (36)	28 (35.4)	—	9 (39.1)	27 (36.0)	—
L_5_–S_1_	7 (63.6)	24 (57.1)	—	14 (56)	34 (43.1)	—	12 (52.2)	36 (48.0)	—
L_5_–L_6_	—	—	—	—	3 (3.8)	—	—	—	—
L_6_–S_1_	—	—	—	—	5 (6.3)	—	—	3 (4.0)	—

### Total disk replacement

**Figure 2.**
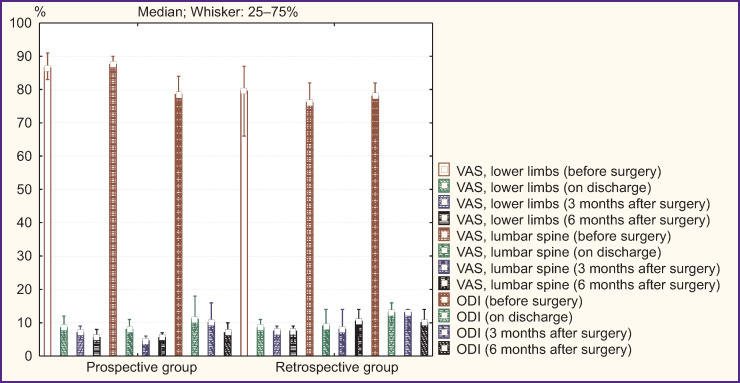
Clinical parameters in patients’ groups under study after lumbar total disk replacement

An intergroup comparison of clinical parameters in surgical treatment of patients with artificial IVD prostheses according to VAS and ODI revealed a comparable pain syndrome level and a functional status before surgery, on discharge and 3 months later (p>0.05). In addition, 6 months after surgery in a prospective group there were recorded the best clinical outcomes: decreased pain intensity in lower limbs (p=0.02) and in the lumbar spine (p=0.03), an increased functional status by ODI (p=0.02) (Figure 2).

### Minimally invasive rigid stabilization

**Figure 3.**
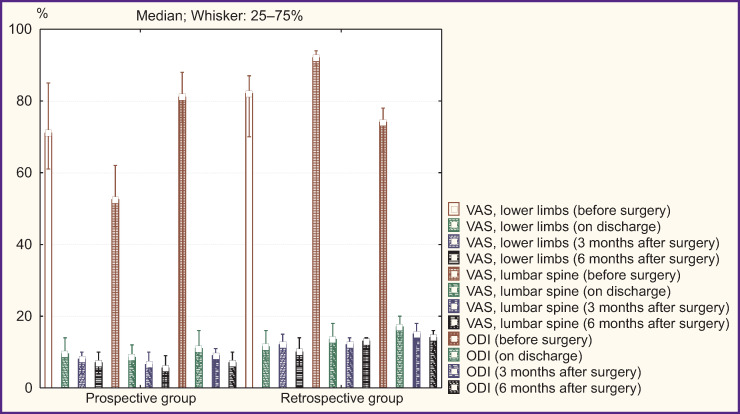
Clinical parameters in patients’ groups under study after minimally invasive transforaminal interbody fusion

An intergroup comparison of clinical parameters in patients after MI-TLIF according to VAS and ODI found a comparable pain syndrome level and a functional status before surgery and on discharge (p>0.05). At terms of 3 and 6 months after surgery, the prospective group was found to have the best clinical outcomes: decreased pain intensity in lower limbs (p=0.01 and p=0.01, respectively) and in the lumbar spine (p=0.03 and p=0.02, respectively), an increased functional status by ODI (p=0.01 and p=0.03, respectively) (Figure 3).

### Open rigid stabilization

**Figure 4.**
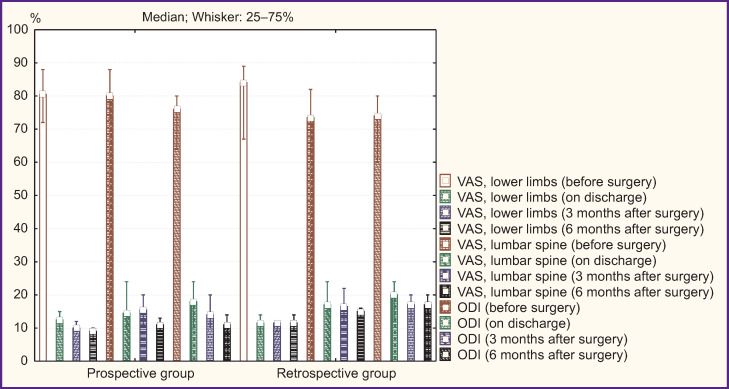
Clinical parameters in patients’ groups under study after open transforaminal interbody fusion

An intergroup comparison of clinical parameters of the patients after O-TLIF according to VAS and ODI revealed a comparable pain syndrome level and a functional status before surgery, on discharge and 3 months later (p>0.05). In addition, 6 months after surgery in the prospective group there were recorded the best clinical outcomes: decreased pain intensity in lower limbs (p=0.04) and in lumbar spine (p=0.03), an increased functional status by ODI (p=0.01) (Figure 4).

## Discussion

Clinical decision support system refers to predictive methods for postoperative periods considering the elimination of modifiable factors, which have an effect on poor result formation [10]. The present approach suggests using ML and AI, the implementation of which in medical practice is promoted by rapid development of computer technologies [13]. ML and AI aim at objectivization and improvement of treatment results, determination of complication probability, and reduction of the number of unfavorable events [18, 19].

Currently, in professional literature there are few reports describing the use of ML and AI algorithms in spinal surgery. Using a deep ML technique, Staartjes et al. [20] developed a preoperative prognosis model of probable pain syndrome reduction in the back and legs, as well as functional status increase according to ODI after lumbar discectomy. However, regression models showed the worst efficiency treatment rate for each of clinical outcomes. The implementation of deep learning technology in the study by Wirries et al. [21] contributed to exact prediction of a patient’s functional status by ODI 6 months both after lumbar microdiscectomy and in case of conservative treatment. Using prospective register data (n=635), Siccoli et al. [22] demonstrated a model based on several ML algorithms to enable to provide preoperative planning of the following results: clinical ODI improvement, pain decrease in legs and in spine 12 months after surgery — within the accuracy of 62, 74, and 66%, respectively; reduction of the total number of reoperations — within the accuracy of 69%, and surgery time — within the accuracy of 78%; the reduced length of hospital stay — within the accuracy of 77%.

ML and AI technologies facilitate in solving narrow-specific tasks. Lee et al. [23] reported that the efficiency of using ML algorithms for exact prognoses of spinal pelvic compensation after fusion to reduce proximal anterior curvature formation risks. Studying the findings of MI surgical techniques (ALIF, XLIF) and conventional TLIF, Campagner et al. [24] showed ML capabilities for preoperative prognosis of invasion degree considering inflammatory markers in patients’ blood. Raman et al. [25] found that using ML technologies enables to reveal blood loss risk factors during surgery and perioperative concentrated red cell transfusion. The authors referred to the stabilization of over 13 spinal segments above 1 degree on ASA scale, three-column osteotomy, and pelvic fixation.

ML and AI implementation in spinal surgery will enable to reduce costs on patients’ treatment [26] and contribute to saving labor time of many related specialists participating in diagnosis, operative treatment, rehabilitation alongside with an operating surgeon [10, 18].

The major problems of using ML and AI are the complexity of applying mathematical calculations when implementing ML and AI, and an ethic aspect of sharing the responsibility between a doctor and a CDSS developer for undesirable sequelae, as well as possible systemic software errors and patients’ unique characteristics, which potentially can affect the decision making and a result [27, 28].

Currently, when using ML- and AI-based models, there should be a force balance between confidence in machine-created algorithm and proper clinical experience.

The present study found CDSS developed on the basis of surgical approach algorithm depending on a number of individual anatomical characteristics of lumbar motor segments to enable to reduce a pain syndrome in the lumbar spine and lower limbs, recover patients’ activities of daily living, and thereby improve their life quality. The use of a developed electronic checklist is a convenient and easy method to obtain recommendations on patient-specific surgical treatment of patients with degenerative lumbar spine diseases.

### Study limitation

A significant study limitation is its single-center nature, small cohorts of patients under study in a prospective group, and a short follow-up of patients operated using a developed CDSS.

## Conclusion

The study results showed high efficiency of a developed clinical decision support system based on individual biometric parameters of lumbar motor segments for personalized surgical management of patients with degenerative diseases of lumbar spine.

However, it is required to carry out further multi-center studies aimed at estimating various anatomical and morphological, biomechanical, clinical, and instrumental parameters in patients operated using a developed clinical decision support system on a larger cohort of subjects within a long-term follow-up.
